# Markers of remodeling in subcutaneous adipose tissue are strongly associated with overweight and insulin sensitivity in healthy non-obese men

**DOI:** 10.1038/s41598-020-71109-4

**Published:** 2020-08-20

**Authors:** Sissel Åkra, Tonje A. Aksnes, Arnljot Flaa, Heidi B. Eggesbø, Trine Baur Opstad, Ida U. Njerve, Ingebjørg Seljeflot

**Affiliations:** 1 Department of Cardiology, Center for Clinical Heart Research, Oslo University Hospital, Ullevål, Pb 4956 Nydalen, 0424 Oslo, Norway; 2 Section of Cardiovascular and Renal Research, Oslo University Hospital, Oslo, Norway; 3 Section for Interventional Cardiology, Department of Cardiology, Heart-, Lung-, and Vascular-Disease Clinic, Oslo University Hospital, Oslo, Norway; 4 Department of Cardiology, Oslo University Hospital, Ullevål, Oslo Norway; 5 Division of Radiology and Nuclear Medicine, Oslo University Hospital, Oslo, Norway; 6 Faculty of Medicine, University of Oslo, Oslo, Norway

**Keywords:** Gene expression, Biomarkers, Endocrinology

## Abstract

Alteration in extracellular matrix (ECM) in adipose tissues (AT) has been associated with insulin resistance, diabetes and obesity. We investigated whether selected biomarkers of ECM remodeling in AT in healthy subjects associated with the amount and distribution of AT and with glucometabolic variables. Subcutaneous AT and fasting blood samples from 103 middle-aged healthy non-obese men were used. AT gene expression and circulating levels of the biomarkers were quantified. Distribution of AT was assessed by computed tomography, separated into subcutaneous, deep subcutaneous and visceral AT. Insulin sensitivity was measured by glucose clamp technique. Metalloproteinase (MMP)-9, tissue inhibitor of MMP (TIMP)-1 and plasminogen activator inhibitor (PAI)-1 expression in AT correlated significantly to the amount of AT in all compartments (r_s_ = 0.41–0.53, all *p* ≤ 0.01), and to insulin sensitivity, insulin, C-peptide, waist circumference and body mass index (BMI) (r_s_ = 0.25–0.57, all *p* ≤ 0.05). MMP-9 was 5.3 fold higher in subjects with insulin sensitivity below median (*p* = 0.002) and 3.1 fold higher in subjects with BMI above median level (*p* = 0.013). In our healthy non-obese middle-aged population AT-expressed genes, central in remodeling of ECM, associated strongly with the amount of abdominal AT, overweight and insulin sensitivity, indicating AT-remodeling to play a role also in non-obese individuals. The remodeling process seems furthermore to associate significantly with glucometabolic disturbances.

**Trial registration:** ClinicalTrials.gov, NCT01412554. Registered 9 August 2011, https://clinicaltrials.gov/ct2/show/NCT01412554?term=NCT01412554.

## Introduction

The adipose tissue (AT) is now recognized as an active endocrine organ in addition to play a central role in the energy homeostasis^1^. Especially abdominal AT has been reported to be associated with atherosclerosis and type 2 diabetes (T2DM) and insulin resistance^2^. It has been suggested that visceral AT (VAT), which seems to be the most active compartment, accumulates when the subcutaneous AT (SAT) loose its ability to expand and store excess energy^3^. The SAT is separated into superficial SAT (sSAT) and deep SAT (dSAT) layers, discussed to differ metabolically with regards to insulin resistance^2^. It has been reported large individual differences in the ability of the SAT to recruit new adipose cells to undergo adipogenesis when excess fat is to be stored^4^. This reduces adipogenesis has furthermore been associated with insulin resistance and T2DM^4–6^.

In the development of overweight and obesity, AT is modified with hypertrophic adipocytes, infiltration of macrophages and other pro-inflammatory immune cells. In addition, dynamic remodeling of the extracellular matrix (ECM) occurs^7^ , crucial for the expansion of the AT to allow necessary and proper structural changes^5^.

A consequence of obesity is accumulation of ECM with subsequent impairment of adapting to metabolic changes, resulting in reduced glucose uptake^6^ and insulin sensitivity^5^. The pathological remodeling is a complex process, involving a number of proteins of which different collagens, and especially collagen-VI is of great importance^8^. The ECM composition is also modified by degrading enzymes, like matrix metalloproteinases (MMPs), of which MMP-9 is one that are produced and released especially from macrophages, but also from adipocytes^7^. The activity of MMPs including MMP-9, are regulated by tissue inhibitors of MMPs (TIMPs)^9^, and also the serine protease inhibitor, plasminogen activator inhibitor (PAI)-1) plays a role in remodeling by inhibiting urokinase-induced plasminogen activation, important for MMP-9 activation^10,11^. PAI-1 is produced and secreted from AT, and seems to be higher expressed in VAT compared to SAT^12^. There is a body of evidence that PAI-1 levels are increased with abdominal obesity, insulin resistance, metabolic syndrome and T2DM^13,14^. GALECTIN 3 which is present both in adipocytes and infiltrating macrophages^15^ localized in the ECM^16^ seems to be involved in expansion and remodeling of AT by stimulating preadipocytes into mature adipocytes^17,18^. GALECTIN 3 has also recently been related to progression of obesity, insulin resistance and T2DM in humans^19–21^, however, conflicting results have been reported^22^.

Although dysfunction in different compartments of AT and their ECM have been associated with metabolic disturbances, the underlying mechanisms are not well understood.

The aim of the present study was therefore to investigate whether markers of adipose tissue ECM remodeling, genetically expressed in subcutaneous AT, as well as circulating levels, associate with the amount and distribution of abdominal AT, and with glucometabolic variables, in a cohort of healthy non-obese men. Whether the genetic expression in subcutaneous AT was reflected in the circulating levels were further explored.

Our hypotheses were that markers of remodeling, expressed in AT and as corresponding circulating levels were increased with increased amount of AT, and related to any degree of glucometabolic disturbances, including insulin resistance.

## Materials and methods

The present investigation is a study on 103 middle-aged Caucasian men initially recruited 20 years earlier, from the yearly military draft in Oslo and Akershus, Norway, previously described in details^23^.

Fasting venous blood samples were drawn between 8 and 11 AM at the time of follow-up. Conventional routine methods were used for determination of fasting glucose and serum lipids. HbA1c, insulin and C-peptide were determined by Turbidimetric inhibition immunoassay (Roche, Basel, Switzerland), Insulin was measured by dissociation-enhanced lanthanide fluorescence immunoassay (DELFIA) (Perkin Elmer, Waltham, Massachusetts, USA) and Electro chemiluminiscence immunoassay (ECLIA) (Roche Diagnostics), respectively. Additional serum, obtained by centrifugation within one hour at 2,500 × *g* for 10 min, and citrate blood, kept on ice until centrifugation within 1 h, at 2,500 × *g* for 20 min, were kept frozen at − 80 °C until analysis.

Subcutaneous AT was sampled from the gluteal region and immediately stored at − 80 °C until RNA extraction.

### Laboratory analyses

Circulating levels of MMP-9, TIMP-1 and GALECTIN 3 were measured in serum by commercially available enzyme-linked immunosorbent assays (ELISA) (R&D Systems, Inc., 614 McKinley Place NE, Minneapolis, US). PAI-1 activity was determined by the chromogenic assay SPECTROLYSE PAI-1 (REF 101,201) (BioMedica Diagnostics, Stamford, CT, US) in citrated plasma. The inter-assay coefficients of variation (CV) were 5.1%, 1.0%, 0.8% and 13.8%, respectively.

Total RNA was isolated from subcutaneous AT by use of the RNeasy Lipid Tissue Mini Kit and QIAcube according to the manufacturer protocol (Qiagen, GmbH, Hilden, Germany). The quality and quantity (ng/µL) of RNA were determined by the NanoDropTM 1,000 Spectrophotometer (Saveen Werner, Sweden). cDNA was made from equal amount of RNA (5 ng/µl) with qScript cDNA SuperMix (Quanta Biosciences, Gaithersburg, Maryland, USA). Gene expression analyses of MMP-9 (Hs00234579_m1), TIMP-1 (Hs00171558_m1), GALECTIN 3 (Hs00173587_m1) and PAI-1 (Hs01126606_m1) (all Applied Biosystems, Foster City, CA, USA) were performed with real-time PCR on a ViiA 7 instrument (Applied Biosystems) using custom designed TaqMan RT-PCR Arrays. The mRNA levels from the RT-PCR reaction were determined with the ∆∆CT method, normalized to β2-microglobulin (HS99999907_m1) (Applied Biosystems) and related to a reference sample giving relative quantification (RQ)^24^. The CV for the CT values for β2-microglobulin cross all runs for the total cohort was 9.6%.

### Glucose clamp

To assess insulin sensitivity, glucose disposal rate (GDR), was determined by a 120 min hyperinsulinemic isoglycemic glucose clamp, modified after DeFronzo^25^, and used as previously described in details^26^. In brief, fasting glucose value was calculated from the average of three blood glucose values (Accu-Chek Performa, Roche, Mannheim, Germany) in blood drawn from catheterization of the left antecubital vein. A human insulin analog (Insulin Lispro, Eli Lilly, Houten, The Netherlands) (30 IE) was infused at a constant infusion rate at 1 mU/min/ kg body weight. Glucose infusion was started 5 min later with 20 mL/h for 5 min, 30 mL/h the next 5 min and then adjusted according to the blood glucose values measured every 5 min to keep the participants at their fasting isoglycemic level. GDR was calculated from the average glucose infusion rate from the last 20 min of the clamp divided by body weight (mg/kg/min).

### Computed tomography (CT)

The amount and distribution of AT were assessed by CT. The method has previously been described in details^27^. Briefly, the CT-scan was conducted at the level of L3/4, with a Siemens Somatom Sensation 64 CT Scanner (Erlangen, Germany) and the CT parameters were set to 120 kV, 200 mAs and slice thickness of 5 mm. The DICOM images were analyzed with the software program Osirix (Pixmo, Geneva, Switzerland). The amount, in square centimeter (cm^2^) of dSAT and sSAT was assessed by tracking the circumferences divided by a membranous layer (Scarpa`s fascia). The muscle compartments including the spine, were assessed by tracking the circumferences between the dSAT and the inner abdominal wall. VAT was calculated by highlighting the pixels containing fat (− 30 to − 190 Hounsfield Units) after assessing the muscle compartments including the spine, by tracking the circumferences between the dSAT and the inner abdominal wall. A radiologist blinded for the other study results read the CT-scans.

### Statistics

Non-parametric methods were used throughout due to mostly skewed distributed data. Data are presented as median values (25, 75 percentiles) if not otherwise stated. Correlation analyses were performed with Spearmans rho (r_s_), and multiple comparisons were adjusted for by Bonferroni correction, i.e. p = 0.05/number of comparisons in the models, when appropriate. Mann–Whitney U test was used for comparing continuous data between two groups. The cohort consisted of men with similar age, and the investigated metabolic variables were closely inter-correlated (Supplementary Table 1), therefore no adjustments in group comparisons were performed. *p*-values less than 0.05 were considered statistically significant. SPSS version 23 (SPSS Inc., IL, USA) was used for all analyses.

### Ethics approval and consent to participate

The study was approved by the Regional Ethics Committee and written informed consent to participate was collected from all patients. The study was conducted in accordance to the Declaration of Helsinki.

## Results

Characteristics of the study population are shown in Table 1, and as can be seen the mean age was 39.5 years and the measured glucometabolic variables were within normal ranges. As were also the serum lipid levels. Family history of diabetes was present in 20%, and 5% were smokers. Median BMI was 23.5 kg/m^2^ and high BMI was defined as > 26.3 kg/m^2^ (quartile 4). Median GDR was 6.3 mg/kg/min and low GDR was defined as < 3.8 mg/kg/min) (quartile 1).

**Table 1:** Demographic characteristics, laboratory variables, and adipose tissue measures of the total study population (n = 103).

Age (years)*	39.5 (3.5)
Smoking	5 (5%)
Family history of diabetes	20 (20%)
GDR (mg/kg/min)	6.3 (3.8, 9.0)
Fasting Glucose (mmol/L)	5.0 (4.8, 5.4)
HbA1c (%)	5.3 (5.1, 5.5)
Insulin (pmol/L)	44 (28, 64)
C-peptide (pmol/L)	641 (511, 850)
BMI (kg/m2)	23.5 (21.8, 26.3)
Waist circumference (cm)	93.8 (87.7, 102.6)
Total cholesterol (mmol/L)	5.1 (4.5, 5.7)
HDL cholesterol (mmol/L)	1.3 (1.1, 1.6)
LDL cholesterol (mmol/L)	3.4 (2.7, 3.8)
Triglycerides (mmol/L)	0.98 (0.70,1.51)
SBP (mmHg)	118.0 (112.2, 125.0)
DBP (mmHg)	74.5 (69.5, 78.0)
MMP-9 (ng/mL)	247 (185, 320)
TIMP-1 (pg/mL)	143 (131, 162)
PAI-1 (IU/mL)	24.5 (18.2, 34.1)
Galectin-3 (ng/mL)	8.0 (6.3, 10.1)
sSAT, cm^2^	94.5 (67.3, 124.8)
dSAT, cm^2^	84.5 (50.5, 128.8)
VAT, cm^2^	92.6 (61.2, 149.3)

The data are given as proportions or median values (25, 75 percentiles), if not otherwise stated. *mean ± SD. For abbreviations: see text.

All glucometabolic variables, except HbA1c were highly significantly correlated to the amount of AT in all compartments (rs 0.494–0.873) (Supplementary Table 2).

### Correlations between the measured genes expressed in AT and the amount of abdominal AT

Unadjusted coefficients of correlation are shown in Table 2. The expression of MMP-9, TIMP-1 and PAI-1 in AT was significantly correlated to the amount of abdominal AT in all compartments (sSAT, dSAT and VAT) (all p < 0.001), still significant after Bonferroni correction (p < 0.004 by 12 performed comparisons), whereas the expression of GALECTIN 3 did not correlate significantly to any of the AT compartments.

**Table 2:** Uncorrected coefficients of correlations (Spearmans rho) between the measured genes expressed in subcutaneous adipose tissue (AT) and the corresponding circulating markers and the different abdominal adipose tissue compartments assessed by axial CT scan.

	Subcutaneous AT expression				Circulating levels			
	MMP-9	TIMP-1	PAI-1	GALECTIN 3	MMP-9	TIMP-1	PAI-1	GALECTIN 3
sSAT	0.41***	0.37**	0.53***	− 0.08	0.19	0.11	0.39***	0.16
dSAT	0.42***	0.47***	0.52***	− 0.14	0.20	0.07	0.55***	0.18
VAT	0.51***	0.53***	0.50***	− 0.05	0.13	0.12	0.56***	0.03

**p < 0.01, ***p ≤ 0.001.

When stratifying by subjects without (n = 83) and with a family history of diabetes the same picture was seen in those without (Supplementary Table 3a), but not in those with a family history of diabetes.

### Correlations between the measured genes expressed in AT and glucometabolic variables

The expression of MMP-9 and PAI-1 in AT correlated significantly to all the glucometabolic variables (all *p* < 0.05, Table 3), however, for fasting glucose, the significance was lost after Bonferroni correction (p > 0.002 by 28 comparisons). Also TIMP-1 expression correlated significantly to all (*p* < 0.001, corrected p < 0.002), except fasting glucose and HbA1c. The expression of GALECTIN 3 was inversely correlated to insulin and C-peptide (*p* < 0.05, both), but the significance was lost after correction (p > 0.002).

**Table 3:** Uncorrected coefficients of correlations (Spearmans rho) between the measured genes expressed in subcutaneous adipose tissue (AT) and circulating levels and the glucometabolic variables.

	Subcutaneous AT expression				Circulating levels			
	MMP-9	TIMP-1 PAI-1		GALECTIN-3	sMMP-9	sTIMP-1	sPAI-1	sGALECTIN-3
GDR	− 0.51***	− 0.47***	− 0.50***	0.18	0.18	0.11	− 0.59***	0.05
Fasting glucose	0.33**	0.18	0.25*	− 0.16	0.12	0.13	0.28**	− 0.01
HbA1c	0.46***	0.22	0.38***	− 0.23	0.17	0.09	0.18	− 0.11
Insulin	0.43***	0.42***	0.39***	− 0.28*	0.17	0.15	0.55***	0.03
C-peptide	0.47***	0.41***	0.39***	− 0.30*	0.13	0.16	0.59***	− 0.05
BMI	0.45***	0.48***	0.50***	− 0.20	0.11	− 0.01	0.55***	0.04
Waist	0.52***	0.57***	0.56***	− 0.12	0.13	0.05	0.58***	0.11

*p < 0.05, ** *p* < 0.01, ***p ≤ 0.001. For abbreviations: see text.

Stratified by subjects without (n = 83) and with a family history of diabetes the same picture was seen in those without (Supplementary Table 3b), but not in those with a family history of diabetes.

**Figure 1:**
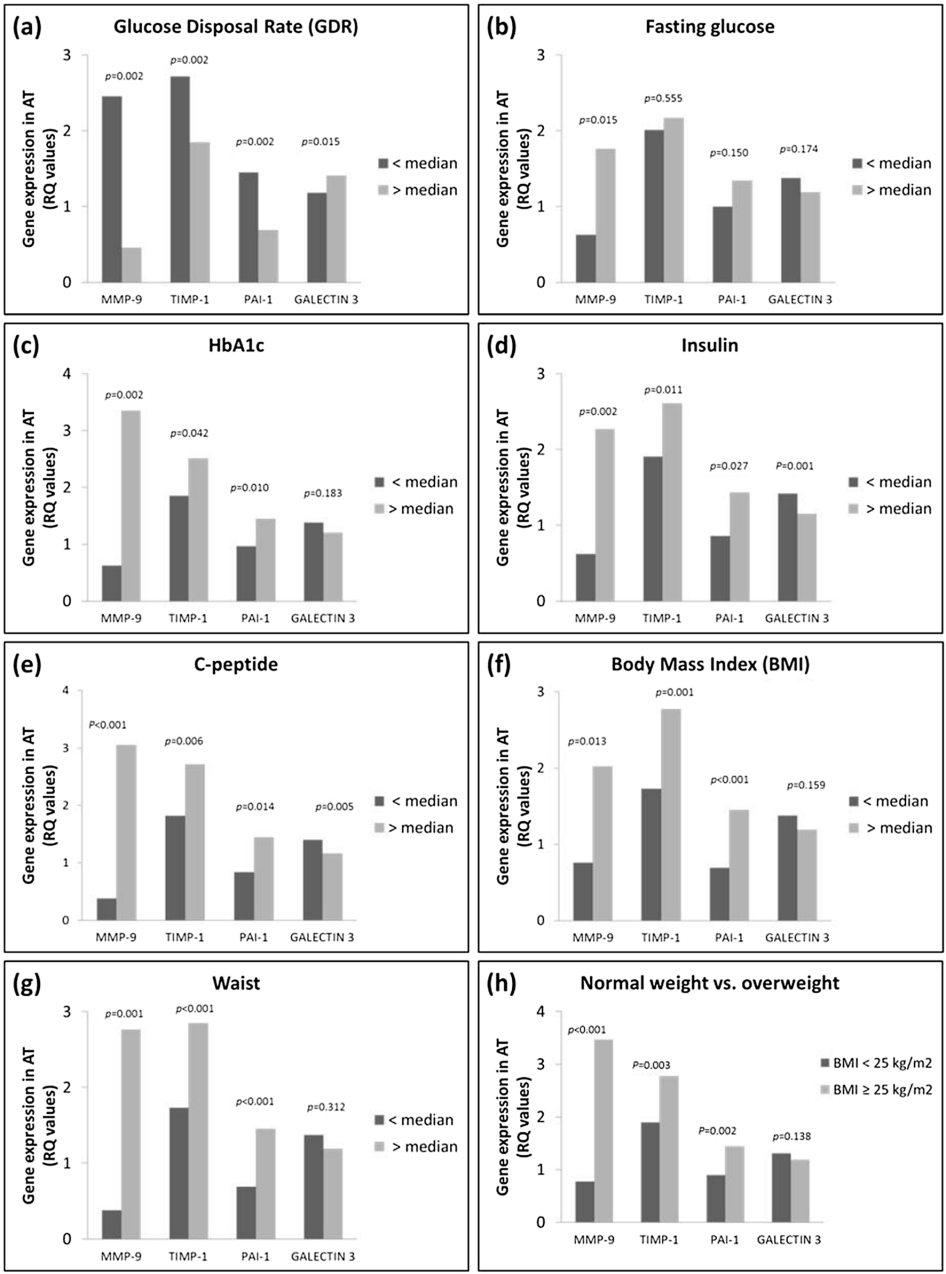
Gene expression of the measured biomarkers in subcutaneous adipose tissue as related to glucometabolic variables dichotomized by median values and as related to being overweight (BMI > 25 kg/m^2^). *p*-values refer to Mann–Whitney U Test.

To further explore the results, glucometabolic variables were dichotomized at the median values (Fig. 1). The group with GDR below median (< 6.3 mg/kg/min) (Fig. 1a) had 5.3 fold higher expression of MMP-9, 1.5 fold higher TIMP-1 and 2.1 fold higher PAI-1 expression (all *p* = 0.002), but significantly lower expression of GALECTIN 3 (1.2 fold) (*p* = 0.015).

In subjects with fasting glucose levels above median (> 5.0 mmol/L), significantly higher expression of MMP-9 was observed (*p* = 0.015), (Fig. 1b), and HbA1c separated by median (5.3%) showed those with levels above median to have significantly higher expression of MMP-9, TIMP-1 and PAI-1 (*p* < 0.05, all) (Fig. 1c). Insulin and C-peptide showed the same pattern with higher expression of MMP-9, TIMP-1 and PAI-1, and were also inversely related to GALECTIN 3 (*p* < 0.05, all) (Fig. 1d,e, respectively).

When separating BMI by median (23.5 kg/m^2^) (Fig. 1f), those with BMI above median had 3.1-fold higher expression of MMP-9, 1.5-fold higher TIMP-1 and 2.1 fold higher PAI-1 expression (all *p* ≤ 0.01), but no difference in the expression of GALECTIN 3. Waist circumference divided by median (93.8 cm), showed the same pattern as BMI (Fig. 1g).

Using the definition of being overweight i.e. BMI ≥ 25.0 kg/m^2^, the same picture was seen with especially MMP-9 to be 5.0-fold higher expressed (Fig. 1h).

**Figure 2:**
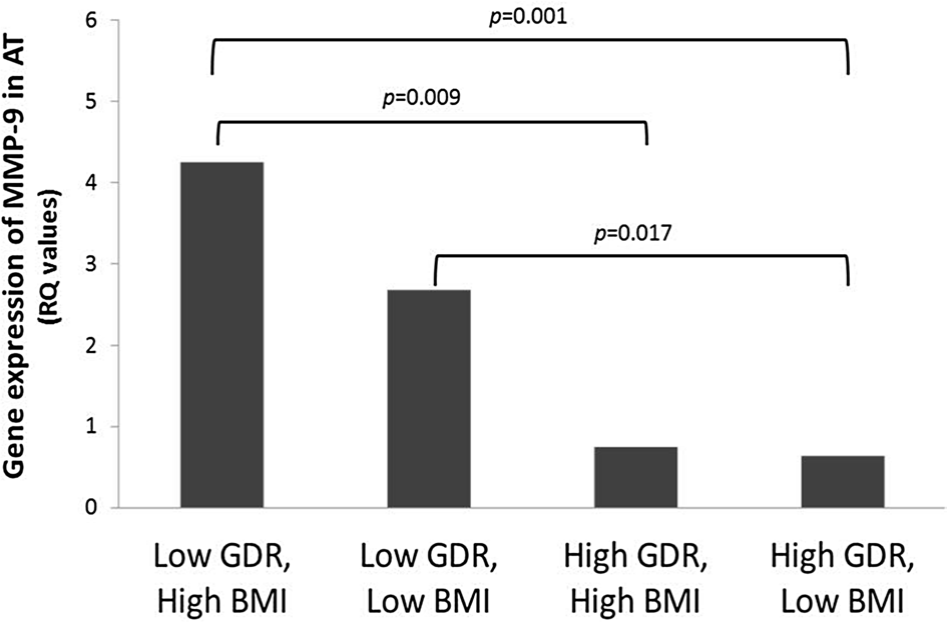
AT gene expression of MMP-9 related to groups combining subjects with low/high GDR and high/low BMI. Low GDR/High BMI: Subjects in the lowest quartile of GDR (< 3.8 mg/kg/min) i.e. with poorest insulin sensitivity and the highest quartile of BMI (> 26.3 kg/m^2^), (n = 11). Low GDR/Low BMI: Subjects in the lowest quartile of GDR and the three lowest quartiles of BMI, (n = 14). High GDR/High BMI: Subjects in the three upper quartiles of GDR (> 3.8 mg/kg/min) and highest quartile of BMI (> 26.3 kg/m^2^), (n = 14). High GDR/Low BMI: Subjects in the three upper quartiles of GDR and the three lowest quartiles of BMI, (n = 59).

When combining groups with high/low BMI separated by quartiles, and low/high GDR separated by quartiles (Fig. 2), it revealed that MMP-9 AT expression was significantly higher in groups with low GDR, either high (p = 0.001) or low (p = 0.017) BMI, compared to the group with high GDR and low BMI. The group with low GDR and high BMI had also significant higher expression compared to the group with high GDR and high BMI (p = 0.009). The lowest expression occurred in the group with the combination of both low BMI and high GDR, i.e. were most insulin sensitive.

### Circulating levels

Measured in the circulation, only PAI-1 correlated significantly to the amount of AT in all compartments (all p ≤ 0.001) (Table 2), still significant after Bonferroni correction (p < 0.004). PAI-1 correlated furthermore to glucometabolic variables (all *p* < 0.05), still significant after correction (p < 0.002), except for HbA1c and fasting glucose, whereas MMP-9, TIMP-1 and GALECTIN 3 did not correlate significantly to any of the glucometabolic variables (Table 3). And as shown in Table 4, PAI-1was also the only marker found to correlate significantly to its corresponding AT gene expression (*p* < 0.001, corrected p = 0.004).

**Table 4:** Uncorrected coefficients of correlations (Spearmans rho) between the measured genes expressed in subcutaneous adipose tissue (AT) and corresponding levels in circulation (s).

Gene expression	Circulating levels			
	sMMP-9	sTIMP-1	sPAI-1	sGALECTIN 3
AT MMP-9	− 0.037	0.072	0.354**	0.051
AT TIMP-1	0.039	0.099	0.534***	0.030
AT PAI-1	− 0.023	0.247*	0.425***	0.044
AT GALECTIN 3	− 0.051	− 0.260*	− 0.056	0.084

*p < 0.05, **p < 0.01, ***p < 0.001.

## Discussion

In this study on healthy men without metabolic disease we observed that: (1) gene expression in subcutaneous AT of MMP-9, TIMP-1 and PAI-1, reflecting ECM remodeling, strongly associated with the amount of abdominal AT, assessed by CT, (2) the expression of these remodeling markers was strongly associated with glucometabolic variables including insulin sensitivity and BMI, (3) measured in the circulation, only PAI-1 correlated to the amount of AT and to the corresponding gene expression, and (4) PAI-1 was also the only marker measured in the circulation which correlated significantly to any of the glucometabolic variables. Interestingly, when excluding the subset of subjects having a family history of diabetes, these associations were even strengthened.

It is well known that VAT is inflamed, especially in obesity and in subjects with the metabolic syndrome and in patients with atherosclerotic coronary artery disease, and is capable of secreting large amount of pro-inflammatory and atherogenic substances^28^ . Although the genes in our study were measured in subcutaneous AT, similar associations were observed in all compartments i.e. sSAT, dSAT and VAT, suggesting the genes measured in subcutaneous AT to reflect an ongoing overall remodeling of the AT. MMP-9 has previously, in a small study, been shown to be higher expressed in VAT in obese compared to lean individuals^29^, but also to be reduced in subcutaneous AT after weight loss in obese patient with metabolic syndrome^30^. We have recently published data from the present population, were we observed strong associations between the amount of AT and gene expression of the pro-inflammatory cytokine interleukin-18 and the NLR family pyrin domain containing-3 inflammasome (NLRP3) and further between these inflammatory markers and the glucometabolic state^31^. Taken together with the present findings, it seems that elevated amount of AT in non-obese healthy individuals, is accompanied by increased upregulation of inflammation and remodeling, and further reflected in the glucometabolic state, as also shown in obese subjects^32^.

Gene expression of MMP-9 and PAI-1 correlated significantly to all the measured glucometabolic variables, including insulin sensitivity assessed by glucose-clamp, waist circumference and BMI. A similar pattern was observed for TIMP-1, which, in addition to be an inhibitor of MMPs, also has been shown associated with tissue remodeling^33^. The results were strengthened when dichotomizing the glucometabolic levels at median values, showing MMP-9 expression especially, to be significantly higher with less favorable values for all the glucometabolic variables. Our results are supportive to a study on non-diabetic subject, which also showed reduction in subcutaneous AT MMP-9 expression with improved insulin sensitivity after treatment with insulin-sensitizing drugs^32^. In line with our results, it was recently also shown that expression of MMP-9 and TIMP-1 in AT from healthy overweight and obese individuals were associated with BMI, and especially MMP-9 was also strongly related to glucose clamp assessed insulin sensitivity, independent of BMI^34^. The authors discusses and concluded that the remodeling process together with adipogenesis in AT are the initial processes leading to reduced insulin sensitivity. This hypothesis is even strengthened by our additional findings of the strong associations with the amount of AT assessed by CT and further, that MMP-9 expression was high in individuals with poor insulin sensitivity, either high or low BMI. With our cross-sectional approach any course or consequence cannot be discussed, however, as shown (Supplementary Table 2), increased fat mass in all compartments associated with lower GDR, i.e. that fat mass may be the driver of reduced GDR, and that the ECM modifiers have only indirect relationship to GDR, via increased fat mass.

GALECTIN 3 expression did not correlate to BMI, but was inversely related to insulin sensitivity, insulin and C-peptide, although vanished after Bonferroni correction. This is to some degree in line with results from an animal model showing that GALECTIN 3 deficiency mice fed on a high fat diet develop increased VAT and increased fasting glucose, HbA1c and insulin resistance, indicating GALECTIN 3 to be protective against T2DM and obesity^35^. In another study increased accumulation of advanced glycation end products (AGEs) in the pancreatic islets was shown with GALECTIN 3 deficiency, suggesting GALECTIN 3 also to play a protective role against AGE related injury^36^.

The only significant association found between the measured markers in circulation and the corresponding gene expression in AT was for PAI-1, which is in line with others studies, suggesting AT to be a major source of circulating PAI-1^37,38^. These findings are further supported in our study by PAI-1 being the only circulatory marker that also correlated to the amount of abdominal AT, in all compartments. PAI-1 was also the only circulatory marker that associated significantly to the glucometabolic variables, which is in line with accepted knowledge^39^. Thus, our hypotheses that the circulating levels of the biomarkers would mirror the corresponding genes expressed in AT and the amount of AT, were only very limited fulfilled.

The lack of correlation between circulating MMP-9, TIMP-1 and GALECTIN 3 and their levels of gene expression in AT as well as the amount of AT, indicates that AT is not the main source for these circulatory levels, which also has been suggested by others^30^, and enlargement of AT per se seems thus not to affect the levels of these biomarkers in the circulation. It should also be emphasized that although the remodeling markers were upregulated at the gene level, not all proteins undergo the post-transcriptional changes mandatory to function as an active protein^40^. Nevertheless, these results indicate that dysregulation in AT, shown by genetic upregulation of the remodeling markers, results in a non-beneficial glucometabolic state, in our healthy non-obese individuals. This hypothesis might be due to reduced storage capacity and reduced/dysfunctional glucose receptors, as also suggested by others^41–44^. It may, however, also be vice versa, that a dysregulated glucometabolism may result in non-beneficial AT remodeling.

Although some previous studies have shown associations between obesity, remodeling of AT, systemic inflammation and insulin resistance, our results contribute to elucidate early changes in AT also in healthy individuals, to be strongly association with insulin resistance and overweight. This is further underscored by the findings that those having a family history of diabetes, thus, probably already having more disturbed glucometabolism, no such associations were found. The number of subjects in this group was small and the results have to be confirmed in other studies.

### Limitations

Our study is limited by its cross-sectional design and that only healthy men are included, and the results can therefore not be generalized. It nevertheless, supports experimental studies in this field^45^. A limitation is also that we used subcutaneous adipose tissue samples as sampling from other anatomic areas was not feasible. The results can therefore not be directly translated to adipose tissue in general. The markers investigated selected out of the complex process of remodeling, involving a number of proteins including collagens, can be discussed. The selection was based to elucidate corresponding proteins at a measurable level in the circulation, as indicated in our hypothesis. It should also be emphasized that our results occur from adipose tissue, not distinguished between adipocytes and macrophages.

Strengths in our study are CT-measurement of AT amount in different compartments, and the measure of insulin sensitivity performed by glucose clamp technique.

## Conclusion

In our healthy non-obese population, AT-expressed genes central in remodeling of ECM, were strongly associated with overweight, insulin sensitivity and the amount of abdominal AT, indicative of remodeling to be important in adipose tissue also in non-obese individuals. The remodeling process and glucometabolic disturbances seem to be mutually affected, however, any course or consequence cannot be drawn from this cross sectional study, and has to be further explored.

## Supplementary information

Supplementary file1

## Data Availability

Data is available from the authors by request.
