# Supplementary material for: Markers of remodeling in subcutaneous adipose tissue are strongly associated with overweight and insulin sensitivity in healthy non-obese men
Source: Sci Rep. 2020 Aug 20;10:14055. doi: 10.1038/s41598-020-71109-4 (PMC7441176; doi:10.1038/s41598-020-71109-4)
Supplement: Supplementary file 1 — Supplementary file1 [file 41598_2020_71109_MOESM1_ESM.docx]

**Title of the manuscript:**

Markers of remodeling in subcutaneous adipose tissue are strongly associated with overweight and insulin sensitivity in healthy non-obese men

**Author list:**

1. Sissel Åkra*
2. Tonje Aksnes
3. Arnljot Flaa
4. Heidi Eggesbø
5. Trine Opstad
6. Ida Njerve
7. Ingebjørg Seljeflot

Supplementary Table 1. Uncorrected inter-correlations (Spearmans rho) between the glucometabolic variables

|  | GDR | Fasting glucose | HbA1c | Insulin | C-peptide | BMI | Waist |
| --- | --- | --- | --- | --- | --- | --- | --- |
| GDR | - | -0.391*** | -0.242* | -0.719*** | -0.676*** | -0.637*** | -0.670*** |
| Fasting glucose |  | - | 0.321*** | 0.408*** | 0.443*** | 0.388*** | 0.405*** |
| HbA1c |  |  | - | 0.183 | 0.205* | 0.214* | 0.198* |
| Insulin |  |  |  | - | 0.921*** | 0.684*** | 0.733*** |
| C-peptide |  |  |  |  | - | 0.655*** | 0.730*** |
| BMI |  |  |  |  |  | - | 0.936*** |
| Waist |  |  |  |  |  |  | - |
| **p*<0.05, ****p*≤0.001 | | | | | | | |

Supplementary Table 2. Correlations (Spearmans rho) between the amount of AT in the different compartments and the glucometabolic variables

|  | sSAT | dSAT | VAT |
| --- | --- | --- | --- |
| GDR | -0.524*** | -0.670*** | -0.635*** |
| Fasting Glucose | 0.213* | 0.329*** | 0.462*** |
| HbA1c | 0.137 | 0.160 | 0.179 |
| Insulin | 0.560*** | 0.710*** | 0.700*** |
| C-peptide | 0.494*** | 0.664*** | 0.681*** |
| BMI | 0.770*** | 0.873*** | 0.743*** |
| Waist | 0.802*** | 0.913*** | 0.834*** |

*p<0.05; ***p<0.001

Supplementary Table 3. Uncorrected coefficients of correlations (Spearmans rho) between the measured genes expressed in subcutaneous adipose tissue (AT) and the corresponding circulating markers, and the different abdominal adipose tissue compartments (a) and between the genes expressed in AT and circulating levels, and the glucometabolic variables (b) in subjects without a family history of diabetes (n=83)

| a) |  | Gene | expression |  |  |  | Circulating | levels |  |
| --- | --- | --- | --- | --- | --- | --- | --- | --- | --- |
|  | MMP-9 | TIMP-1 | PAI-1 | GALECTIN 3 |  | MMP-9 | TIMP-1 | PAI-1 | GALECTIN 3 |
| sSAT | 0.50*** | 0.42** | 0.65*** | 0.00 |  | 0.12 | 0.17 | 0.44*** | 0.14 |
| dSAT | 0.48*** | 0.46*** | 0.64*** | -0.02 |  | 0.11 | 0.14 | 0.50*** | 0.17 |
| VAT | 0.58*** | 0.48*** | 0.53*** | -0.107 |  | 0.07 | 0.21 | 0.51*** | 0.04 |
| GDR | -0.51*** | -0.53*** | -0.54*** | 0.13 |  | -0.17 | -0.19 | -0.59*** | 0.05 |
| b) |  |  |  |  |  |  |  |  |  |
| F.glucose | 0.24* | 0.16 | 0.13 | -0.07 |  | 0.05 | 0.13 | 0.19* | -0.01 |
| HbA1c | 0.32* | 0.07 | 0.27** | -0.30 |  | 0.16 | 0.10 | 0.26* | -0.11 |
| Insulin | 0.45*** | 0.41*** | 0.34*** | -0.19 |  | 0.10 | 0.15 | 0.54*** | 0.03 |
| C-peptide | 0.47*** | 0.38*** | 0.31*** | -0.15 |  | 0.05 | 0.16 | 0.58*** | -0.05 |
| BMI | 0.48*** | 0.50*** | 0.56*** | -0.13 |  | 0.05 | 0.03 | 0.52*** | 0.04 |
| Waist | 0.57*** | 0.57*** | 0.62*** | -0.03 |  | 0.11 | 0.10 | 0.55*** | 0.11 |

*p<0.05; **p<0.01; ***p<0.001
